# Comparison of some economic traits by genetic cluster of Aberdeen Angus cattle

**DOI:** 10.5194/aab-68-279-2025

**Published:** 2025-05-12

**Authors:** Judit Márton, Szabolcs Bene, István Anton, Attila Zsolnai, Ferenc Szabó

**Affiliations:** 1 Hungarian Hereford, Angus and Galloway Breeders Association, Kaposvár, Hungary; 2 Institute of Animal Sciences, Georgikon Campus, Hungarian University of Agriculture and Life Sciences, Keszthely, Hungary; 3 Institute of Animal Sciences, Kaposvár Campus, Hungarian University of Agriculture and Life Sciences, Kaposvár, Hungary; 4 Department of Animal Sciences, Albert Kázmér Faculty of Mosonmagyaróvár, Széchenyi István University, Győr, Hungary

## Abstract

The Angus cattle population of Hungary was categorized into four groups using 12 microsatellite markers exhibiting notable genetic variations. Moreover, some traits influencing the profitability and sustainability of beef cattle farming were compared between groups. Data were obtained from 5075 cows (born between 1990–2020) and 19 142 calves (born between 1997–2023), including 10 629 bull calves and 8513 heifer calves of different genetic backgrounds. Genetic groups were distinguished by origin, color, size, and type: blue group (BG), red group (RG), green group (GG), and yellow group (YG). The six investigated traits were age at first calving (AFC), productive lifespan (PL), number of calves born (NCB), culling age (AGE), birth weight (BW), and the 205 d adjusted weaning weight (WW). The averages of the six tested traits were as follows: AFC – 2.35 ± 0.54 years; NCB – 5.89 ± 3.69 heads; PL – 6.85 ± 4.13 years; AGE – 9.2 ± 4.26 years; BW – 29.4 ± 4.28 kg; and WW – 176.9 ± 44.07 kg. YG excelled in terms of NCB, PL, AGE, and WW traits, while RG performed best in terms of AFC and BW. BG displayed the lowest performance across NCB, PL, AGE, BW, and WW. The heavier Red Angus individuals were largely behind the performance of the traditional-type, smaller British-type Black and Red Angus individuals in the tested traits affecting sustainability and profitability. Significant reproduction and longevity trait differences exist among different genetic groups of Angus beef cattle genotyped by DNA microsatellite information. The results indicated significant differences in terms of the performance of different Angus types with regard to the tested traits. These findings could be useful in developing breeding concepts and making selection decisions, contributing to more efficient and sustainable breeding strategies.

## 1 Introduction and literature review

Beef cattle farming, including the Angus breed, is an important global source of quality beef. To understand the economy and sustainability of beef cattle farming, the reproductive and lifespan traits of cows and the growth potential of their calves are very important.

Szabó et al. (2013) have found in their studies that, if the economic weight of the 205 d weaning weight is considered to be 100 %, the relative values of some other traits are as follows: the conception rate of cows equates to 190 % to 770 %, the productive lifetime of cows equates to 50 % to 500 %, calf weight at 120 d of age equates to 70 % to 180 %, the loss of calves at birth equates to 6 % to 170 %, and the conception rate of heifers equates to 40 % to 160 %. Pulina et al. (2021) have indicated that the main limitations of the beef sector are unfavorable reproduction, narrower meat output per live weight, significantly lower productivity, and a longer production cycle compared to other animal species. According to Boyer et al. (2020), six weaned calves were needed to recoup the investment cost of a beef cow. When the economic sustainability of beef cattle farming is considered, reproductive performance is a decisive issue. Increasing efficiency, reducing dependence on subsidies, improving profitability, and meeting social expectations of sustainable beef production are key to the long-term viability of the sector.

The sustainability of the cow–calf operation depends largely on the longevity and the first calving age of cows (Damiran et al., 2018a). Increasing the lifespan directly improves profitability and reduces costs. The length of time spent in production indirectly indicates animal health, resilience, and well-being (Oliveira et al., 2020). The most sustainable beef cattle are cows with lower mature body weight and weaned calves with relatively higher weight (Snelling et al., 2022). The brood cows' demand for feed and resources is lower, their environmental burden is lower, they have more favorable reproductive characteristics, their productive lifespan is longer, and they are better adapted to extreme climatic conditions. The effectiveness of beef cattle farming and breeding depends, to a large extent, on the productive life of cows (Cundiff et al., 1992).

Since the Angus breed plays an important role in beef production, attention should be paid to this breed. The Black and Red Angus breeds are considered to be close relatives due to their low genetic distance. However, based on the research of Kuehn (2010), genetic differences can be observed between them. The mature weight of the cow is a well-heritable trait, with h^2^ = 0.55 to 0.85 (Phillips and Coventry, 2004), and can be easily changed through selection.

With time, beef and Angus herds in the United States have steadily increased in size. The additional cost of maintaining a higher body weight is not compensated for by the increased weight of the calves, the income from culled cows, and the environmental burden. Meanwhile, the greenhouse gas emissions are more significant (Wiseman et al., 2018). DeVuyst et al. (2022) have investigated the profitability differences of Angus, Red Angus, and Lowline Angus. These three types come from the same lineage but have evolved under different selection pressures. The Angus was imported from Scotland to Kansas, USA, in 1873. The Red Angus color is a result of a pigmentation caused by a recessive gene, and the Lowline (lower growth rate) is the consequence of selection made at Trangie Agricultural Research Center (Australia). The highest yield of results was achieved with smaller cows inseminated with Red Angus bulls. These animals had the heaviest calf weaning weight compared to cow weight and the lowest cow maintenance cost.

The ancestors of the current Angus stock in Hungary came from 60 pregnant Aberdeen Angus heifers imported from England in 1980 and the reproductive material of 300 American Red Angus imported in the same year. In 1994, new breeds were established with Canadian Red Angus embryos and semen, as well as German Red Angus imports. The American, Canadian, and British lines were primarily used for breeding.

In our previous study (Márton et al., 2024), the Angus population was genetically grouped based on microsatellite markers. Various groups have indicated some phenotypic differences. However, there is little information about the most important traits of different Angus genetic groups. Therefore, the objective of this study was to analyze the selective traits of different genetic groups of the Angus breed that determine sustainability and profitability. We were curious to see the age at first calving, productive lifespan, number of calves born, culling age, 205 d adjusted weight, and birth weight of the different groups of the Aberdeen Angus breed, separated based on their DNA microsatellite markers.

## 2 Materials and methods

### 2.1 Source of data and the investigated cattle herds 

The data for the study were obtained from the cattle pedigree and registration database of the Hungarian Hereford, Angus, Galloway Association. Individuals with pedigree Aberdeen Angus and a high ( > 75 %) Aberdeen Angus blood proportion were selected from the 16 herds in the study.

The studied population consisted of 5075 cows (born between 1990 and 2020) and 19 142 calves (born between 1997 and 2023; 10 629 bull calves and 8513 heifer calves).

The six investigated traits were age at first calving (AFC), productive lifespan (PL), number of calves born (NCB), culling age (AGE), birth weight (BW), and 205 d adjusted weaning weight (WW).

For the PL, the period from the birth of the first calf born alive to the culling of the cow was considered. For the AGE, individuals that were sold for further maintenance and removed from the association's database were not considered. When determining the NCB and the date of the first calving, only live calves were taken into account. Abortions and still-births were ignored. The BW is measured within 24 h of calving, and the weaning weight is measured individually when the calves are 6 to 9 months old. The WW was calculated as follows:

1 $$\mathrm{WW}=\left(\frac{\mathrm{CW}-\mathrm{BW}}{\mathrm{WD}}\right)\times 205+\mathrm{BW},$$

where WW denotes calf adjusted 205 d weaning weight (kg), CW denotes calf weaning weight (kg), BW denotes calf birth weight (kg), and WD denotes calf weaning day (day).

### 2.2 The genetic groups

The genetic structure and characteristics of the Hungarian Angus cattle population (Tables 1, 2) were studied using 12 microsatellite markers (BM1824, BM2113, ETH3, ETH10, ETH225, INRA023, TGLA122, TGLA126, BM1818, MGTG4B, CSSM66, and CSRM60) detected by an automated ABI 3500 Genetic Analyzer (Applied Biosystems, Foster City, CA, USA). Principal coordinate analysis, assignment tests, and dendrograms all suggest that there are mainly four different groups among Hungarian Angus herds. Structure analysis has yielded K= 4 as the most probable number of clusters. The collection of blood samples was an integral part of the regularly executed routine parentage testing performed by trained veterinarians. The genetic grouping of the studied Angus population was based on DNA microsatellite-based identification (Márton et al., 2024).

**Table 1:** Genetic group characteristics.

Herd	Sires used for insemination	Color	Body size/type
A	exclusively large-framed Red Angus from Canadian and American lines	red	modern, large-framed
C	exclusively large-framed Red Angus from Canadian and American lines	red	modern, large-framed
M	Red Angus from C line	red	modern, large-framed
B	Black Angus from D line, traditional British type	black	traditional, British type
D	black: imported semen from England; red: traditional-type Red Angus from England, American semen	70 % black, 30 % red	traditional, British type
E	Black Angus from D line, traditional British type	80 % black, 20 % red	traditional, British type
G	Black Angus from D line, traditional British type, Black Angus from Austria	black	traditional, British type
H	Black Angus from D line, traditional British type	black	traditional, British type
I	Black Angus from D line, traditional British type	black	traditional, British type
F	Red Angus bulls from D herd	red	traditional, British type
J	traditional-type Red Angus from D line, Red Angus from other traditional herds	red	traditional, British type
L	Red Angus from D line	red	modern, large-framed, traditional British type
N	Red Angus from C line	red	modern, large-framed, German Angus
O	Red Angus from C and D lines	red	modern, large-framed, traditional, German Angus
P	German Red Angus	red	German Angus
K	Red Angus from D and other traditional herds	red	traditional, British type

**Table 2:** Descriptive statistics of age, lifespan, and number of calves of Angus groups.

Genetic group	Statistics	AFC	NCB	PL	AGE
BG	N	2504	2504	2504	2504
	Mean	2.31	5.30	6.14	8.44
	Median	2.06	5.00	5.88	8.30
	SD	0.50	3.60	4.13	4.11
	SE	0.01	0.07	0.08	0.08
	Minimum	1.34	1	0.00	1.92
	Maximum	4.36	16	18.73	20.70
RG	N	468	468	468	468
	Mean	2.28	5.81	7.44	9.73
	Median	2.14	6.0	7.62	9.88
	SD	0.49	3.31	4.52	4.57
	SE	0.02	0.15	0.21	0.21
	Minimum	1.28	1	0.01	1.69
	Maximum	4.56	14	19.08	21.02
GG	N	1988	1988	1988	1988
	Mean	2.40	6.49	7.44	9.85
	Median	2.15	6.00	7.42	9.89
	SD	0.57	3.70	4.14	4.14
	SE	0.01	0.08	0.09	0.09
	Minimum	1.07	1	0.00	1.82
	Maximum	4.54	18	18.71	21.54
YG	N	115	115	115	115
	Mean	2.47	8.85	9.80	12.27
	Median	2.22	10.00	10.60	13.72
	SD	0.68	4.15	4.61	4.53
	SE	0.06	0.39	0.43	0.42
	Minimum	1.31	1	0.01	1.86
	Maximum	4.53	15	19.13	21.15
Total	N	5075	5075	5075	5075
	Mean	2.35	5.89	6.85	9.20
	Median	2.09	6.00	6.84	9.21
	SD	0.54	3.70	4.25	4.26
	SE	0.01	0.05	0.06	0.06
	Minimum	1.07	1	0.00	1.69
	Maximum	4.56	18	19.13	21.54

AFC denotes age at first calving, NCB denotes number of calves born, PL denotes productive lifespan, AGE denotes culling age, BG denotes blue group, RG denotes red group, GG denotes green group, and YG denotes yellow group.

Four genetically defined groups were identified by different colors and population codes from the 16 Hungarian Angus herds: blue group (BG) (ACM groups), green group (GG) (BDEGHI groups), red group (RG) (FJLNOP groups), and yellow group (YG) (K group).

In the blue group (BG), herd A was made up of Canadian and American lines of large-framed Red Angus, crossbred with Canadian-type Red Angus from 1996 (R1, R2, R3, R4 genotype); herd C was made up of embryos from Canadian Red Angus, used as genetic material since 1994, alongside upgrading; and herd M was made up of pregnant Red Angus heifers from herd C in 2011.

In the green group (GG), herd B was made up of pregnant Black Angus heifers derived from E and G lines in 2010 as founder animals, crossed with traditional British-type Black Angus from the D line; herd D was initiated in 1980 with 60 pregnant Aberdeen Angus heifers imported from England, crossbred with traditional-type bulls from Blonde d'Aquitaine and Red Angus imported from England and America; herd E was made up of pregnant heifers from herd D in 1996; herd G was made up of pregnant heifers from herds D and E in 1998; herd H was made up of pregnant heifers from herd D in 2002; and herd I was made up of pregnant heifers from herd D in 2003.

In the red group (RG), herd F was made up of pregnant Red Angus heifers from herds A and D and other Hungarian traditional-type herds in 2011; herd J was made up of pregnant Red Angus heifers from herd D in 2004, Red Angus bulls from herd D and other traditional herds, and Red Angus bulls from herd K; herd L was made up of pregnant Red Angus heifers from herds C and D in 1998; herd N was made up of Red Angus heifers from herds A and P in 2013; herd O was made up of Red-Angus-sired heifers from herds N and P in 2010; and herd P was made up of German Red Angus imported in 1996.

Finally, in the yellow group (YG), herd K was made up of pregnant Red Angus heifers from herd D in 1998, with less than 5 % Limousin blood proportion.

### 2.3 Statistical analysis

The Kolgomorov–Smirnov test was used to check the normal distribution of the data. The homogeneity of the variances was determined using Levene's test. Based on this test, homogeneous variance was not achieved for any traits.

The groups were compared using the Kruskal–Wallis test, and the differences between the groups were measured using Tamhane's post hoc test. For all statistical analyses, significance was declared at p<0.05 .

The study was performed with SPSS 27.0 (2020) software (IBM Corporation, 2020).

## 3 Results and discussion

Descriptive statistics of the data are shown in Table 2, while Table 3 summarizes the performance of different genetic groups. The examination of the specific production and performance data of the genetic groups was implemented for the following traits: AFC, AGE, BW, NCB, PL, and WW. These all showed significant differences.

**Table 3:** Distribution of mean values of age at first calving (AFC), number of calves born (NCB), productive lifespan (PL), culling age (AGE), birth weight (BW), and calf adjusted 205 d weaning weight (WW) and their SD among the four groups identified by structure clustering.

Traits	BG	RG	GG	YG	Total	p
AFC	^b^2.31 ± 0.50	^b^2.28 ± 0.49	^a^2.40 ± 0.57	^a^2.47 ± 0.68	2.35 ± 0.54	< 0.01
NCB	^d^5.30 ± 3.59	^c^5.81 ± 3.32	^b^6.49 ± 3.70	^a^8.85 ± 4.15	5.89 ± 3.69	< 0.01
PL	^c^6.14 ± 4.13	^b^7.44 ± 4.52	^b^7.44 ± 4.14	^a^9.80 ± 4.61	6.85 ± 4.25	< 0.01
AGE	^c^8.44 ± 4.11	^b^9.73 ± 4.57	^b^9.85 ± 4.14	^a^12.27 ± 4.53	9.20 ± 4.26	< 0.01
BW	^b^29.23 ± 4.20	^a^31.35 ± 5.80	^b^29.25 ± 3.61	^a^30.77 ± 3.81	29.4 ± 4.28	< 0.01
WW	^c^166.57 ± 41.09	^a^211.10 ± 40.28	^b^206.12 ± 36.90	^ab^212.55 ± 25.95	176.90 ± 44.07	< 0.01

BG denotes blue group, RG denotes red group, GG denotes green group, and YG denotes yellow group; treatments without the same superscript differ significantly ( p<0.05 ).

### 3.1 Age at first calving (AFC)

The value of AFC differed slightly for the studied groups (Table 3). The average AFC of the four genetic groups was 2.3 ± 0.5 years. The lowest AFC was found in the RG at 2.3 ± 0.5 years, while the highest was in the YG, with a value of 2.5 ± 0.7 years. The difference between the lowest and the highest value was 0.2 years or 73 d. The differences between the genetic groups for the AFC were significant ( p<0.01 ). During their investigation of the AFC of different beef cattle, Dákay et al. (2006) found that the AFC of Aberdeen Angus cows was 2.76 years, while the average AFC of Czech Angus heifers was 2.07 years (Brzáková et al., 2020). Compared to the Hungarian tests of Dákay et al. (2006), 0.41 years (149.8 d) is a significant development, but it is still small compared to the ideal of 2 years (24 months).

Research by Byrne et al. (2022) has shown that a heifer calving at 36 months compared to 24 months consumes 65 % more grass, 96 % more silage, and 33 % more concentrate. At first calving, an AFC of 23 to 25 months optimizes economic performance, minimizes the non-productive period, and maintains the seasonal calving pattern (Wathes et al., 2014). A low AFC increases productivity and decreases replacement rates (López-Paredes et al., 2018). The profitability and sustainability of the cow–calf operation depend largely on the longevity and the first calving age (Damiran et al., 2018a). The maximum efficiency is achieved when the replacement heifers give their first calf at 24 months without complications, when the cows give birth to a calf every year until the optimal culling age, and when the death rate of the calves is below 5 %. Based on data from the Cattle Tracing System (database for all cattle in Great Britain), 8.5 % of beef heifers in Great Britain give birth before the expected age of 24 months; the average period between calvings for beef cows is 394 d (Gates, 2013). The heifers that give birth earlier in the breeding season stayed in the herd for a longer period, and their calves were weaned with a higher total weight than their counterparts that were born later (Cushman et al., 2013).

### 3.2 Number of calves born (NCB)

The NCB differed significantly among the examined groups (Table 3). The average NCB of the four genetic groups is 5.9 ± 3.7 heads. The differences between the genetic groups based on NCB were significant ( p<0.01 ). The lowest NCB (5.3 ± 3.6 heads) was found in the BG, and the highest value was in the YG (8.9 ± 4.2 heads). The difference between the lowest and the highest value is 3.6 calves. The number of calves directly affects profitability and sustainability, and improving it affects economic competitiveness.

Stewart and Martin (1983) have obtained an NCB of 6.4 ± 0.3 heads based on 12 years of data from 113 Angus cows. The defining element of sustainable beef cattle breeding is the reproductive performance of breeding females. Reproductive traits have 3 to 9 times more influence on profitability and sustainability than other production parameters (Melton, 1995). Boyer et al. (2020) determined the return on investment of the beef cow to be six calves. A cow needs to wean at least five consecutive calves to recoup its investment, which is only achieved by cows that calve early (Damiran et al., 2018b). In order to refund the investment costs of the cow–calf operation, it is necessary to wean at least five or six calves (Kertz et al., 2023). Maximizing the number of calves born and weaned and maternal productivity is a constant challenge for beef farmers (Walmsley et al., 2018).

### 3.3 Productive lifespan (PL)

The PL differed greatly among the studied groups (Table 3). The average PL of the four genetic groups was 6.9 ± 4.3 years. The differences between the genetic groups were significant ( p<0.01 ). The lowest PL was found in the BG (6.1 ± 4.1 years), while the highest value (9.8 ± 4.6 years) was found in the YG. The difference between the lowest and highest values was 3.7 years, a difference of at least three calves, which was confirmed by the NCB data. Dákay et al. (2006) have reported that the PL of Aberdeen Angus cows is 8.28 years. Szabó and Dákay (2009) have determined the longevity to be 8.14 years. Tanida et al. (1988) have found that the average PL of Angus cows was 4.49 ± 0.13 years, and the possibility of genetic improvement of the trait is moderate due to the moderate genetic variance. Calf weaning weight shows a significant positive correlation ( p<0.01 ) with the age of the cow; increasing the productive lifespan of cows might increase the weaning weight of calves. The weaning weight of calves increases continuously for dams aged 2 to 4 years; however, 6- to 8-year-old cows had the largest weaned calves (Wellnitz et al., 2022). These results correspond to the findings of Dákay et al. (2006) and Szabó and Dákay (2009), and there was a regression of 1.43 to 1.29 years in the average of the examined genetic groups (6.9 years).

Cows culled before the age of 5 years cannot fulfill their biological potential. Their weaned calves do not reach the peak weight; moreover, the cost of a replacement heifer is high. Based on the literature, a minimum of five or six weaned calves is needed for the economic return of a cow from the point of view of cost-effective production and economic sustainability (Boyer et al., 2020). Increasing the lifespan directly improves profitability and reduces costs.

### 3.4 Culling age (AGE)

Regarding the examined genetic groups, the AGE (period from birth to culling) differed moderately (Table 3). The average AGE of the four genetic groups was 9.2 ± 4.3 years. The lowest AGE was found in the BG, with a value of 8.4 ± 4.1 years. The highest was found in the YG at 12.3 ± 4.5 years. The difference between the lowest and the highest value was 3.9 years. This difference corresponds to the differences between PL and AFC. The differences between the genetic group AGE values are significant ( p<0.01 ).

Dákay et al. (2006) have compared this trait for different beef cattle breeds. The AGE of Aberdeen Angus cows was 11.03. In Hungarian Angus herds, the proportions remaining in the herd are 68 % at the age of 2 to 4 years and 53 % at the age of 4 to 6 years (Bailey and Mears, 1990). Based on an analysis spanning 23 years, the Angus AGE is 6.68. Tanida et al. (1988), Stewart and Martin (1983) calculated an AGE of 7.4 ± 0.4 based on 12 years of data from 113 Angus cows. The duration for which cows are kept in the herd does not reflect well the genetic and biological characteristics of the herd in all cases; in many cases, this is the decision of the manager and breeder and is influenced by the management and the calf production goals. The probability of remaining in the herd ( p= 0.05) at 5 years was averaged at 69.41 %, and calves weaned from cows aged 8 years or older had a higher total weight than those from 3-year-old dams (Wellnitz et al., 2022). In addition, the pregnancy rate of cows improved for 8-year-old and older animals, which is one of the most important parameters for staying in the herd. Based on these results, the association between genetic groups and AGE reflects the factors influencing the performance of breeding animals and staying in the herd.

### 3.5 Birth weight (BW)

The BW differed slightly in the studied groups (Table 3). The average BW of the four genetic groups was 29.4 ± 4.3 kg. The differences between genetic groups based on BW were significant ( p<0.01 ). The lowest BW was found in the BG (29.2 ± 4.2 kg), and the highest was found in the RG (31.4 ± 5.8 kg). The difference between the lowest and highest BW was 2.2 kg. In Canada, the BW of Angus calves was averaged at 34 kg and was positively correlated with post-weaning daily weight gain (Bailey and Mears, 1990). The average birth weight of Angus heifer calves in Bulgaria is 31.6 kg (Nikolov and Karamfilov, 2020), and an average birth weight of 38.38 kg has been reported for five farms in the Czech Republic (Tomas, 2016). The BW in our study is slightly lower than that reported in the literature references.

### 3.6 Calf adjusted 205 d weaning weight (WW)

The WW differed significantly among the examined groups (Table 3). The average WW of the four genetic groups is 176.9 ± 44.1 kg. The differences between the genetic groups based on WW are significant ( p<0.01 ). The lowest WW was found in the BG (166.6 ± 41.1 kg), and the highest value (212.6 ± 25.9 kg) was found in the YG. The difference between the smallest and the largest weaning weight adjusted to 205 d of age was 46.0 kg. These results are slightly different from the results of the previous study in Hungary; namely, the WW values obtained by us are smaller than the data previously published by Bene et al. (2013). They have published WW for Black Angus calves weighing 217 to 224 kg and for Red Angus calves weighing 210 to 213 kg. The difference can be explained by the fact that, while the data of our results are for the entire Angus herd in Hungary, the data of Bene et al. (2013) came from a single herd from an experimental farm. Since the weaned calf is the only marketable product of a beef cow, the WW is closely related to the profitability of the beef sector and, thus, its sustainability. According to some sources, cows give calves with the highest weaning weight at 6 or 7 years (DeVuyst et al., 2022).

## 4 Conclusions

The results of this study – according to which there are significant differences in terms of the most important reproduction and longevity traits among different genetic groups of Angus beef cattle genotyped by DNA microsatellite information – call attention to a selection possibility. These findings could be useful in the development of breeding programs and may help in making selection decisions, contributing to more efficient and sustainable breeding strategies for the breed. Understanding the relationship between the six traits that significantly influence sustainability and profitability and the different types within the Angus breed can help breeders make more informed decisions. By integrating this knowledge, breeders can prioritize those Angus types with the most positive impact on productivity, profitability, environmental footprint, and sustainability. Further research is needed to explore the interactions between these types.

## Data Availability

The data presented in this study are available on request from the Hungarian Hereford, Angus, and Galloway Breeders Association.
